# Fake science for sale? How *Endocrine Connections* is tackling paper mills

**DOI:** 10.1530/EC-21-0489

**Published:** 2021-09-28

**Authors:** Adrian J L Clark, Simon Buckmaster

**Affiliations:** 1 Editor-in-Chief, Endocrine Connections, Bioscientifica Ltd, Bristol, UK; 2 Senior Publisher, Bioscientifica Ltd, Bristol, UK

One of the major concerns of any scientific journal is that the research it publishes should be reproducible. There have been many alarming estimates for the lack of reproducibility of work published in the biomedical sciences, which has led to widespread trends to require authors to provide more detailed descriptions of methods and reagent sourcing and sharing, a frequent call for deposition of original data in open access databanks, and possibly a greater willingness to publish negative or conflicting results. These efforts are based on the reasonable expectation that the research in question has been performed in good faith.

Very occasionally, one encounters a paper in which some of the data is falsified. This can range from, for example, the relatively benign exclusion of outliers in a dataset for spurious reasons or use of an inappropriate control. Reviewers and editors do their best to identify such occurrences and to remedy them. It has been very rare, however, to encounter entirely falsified manuscripts – until recently.

The pressure to publish for early career scientists and clinicians is intense, and career advancement and income are commonly dependent on this. One should not be surprised, therefore, that the phenomenon of the paper mill has emerged in recent years (1). This is a covert organization that, for a fee, will provide a seemingly credible and data-rich paper, and in some cases will even manage the submission and response to reviewer stages. Several journals have identified these submissions, which are often characterized by a fairly comprehensive series of experiments exploring (typically, but not exclusively) a microRNA and its effect on a biological phenomenon via a specific signaling process. Almost exclusively in our experience, these papers originate from clinical departments in China, with the authors (who inevitably seem to lack institutional email addresses or ORCID iDs) never having published a paper before. This is surprising because the 20 or 30 pieces of data shown in the manuscript figures (immunoblots, cell growth curves, FACS analyses, immunostained sections, etc.) are very clear and highly unlikely to be produced by a busy but laboratory-inexperienced clinician.

This journal has seen tens of such manuscripts submitted this year from authors most probably attracted by our increasing Journal Impact Factor™, the broad spectrum of research covered by the journal and our very inexpensive article publication charges. We believe that we have identified and rejected all of these submissions so far, but this remains a continuing challenge to our editors.

Is there anything we can do to prevent these submissions? In common with most journals, *Endocrine Connections* requires corresponding authors to attest that the submission is original, the authors’ own work and has not been submitted elsewhere. The journals published by Bioscientifica have agreed as a matter of policy that we will henceforth require submitting authors to use an institutional email address unless there is a good reason not to do so. The work reported in the vast majority of biomedical science papers is the product of a research or health provider institution that provides the facilities and/or the patients, and which employs the researchers. Undoubtedly, these organizations would be very unhappy to know that falsified research was being published in their name, and it is entirely reasonable that correspondence about the research should take place through their own email servers and not some personal provider.

It is also useful when screening a new submission to be able to check the author’s previous publication record. While this can usually be accomplished by a quick PubMed search, it is often the case that some surnames are very common and literally hundreds of published authors share the same name and initials. In this case, a unique researcher identifier such as the ORCID iD is extremely valuable, and for this reason we will be asking submitting authors to provide this information on submission of articles.

To enable full scrutiny of submissions, authors submitting to *Endocrine Connections* should know that the editors may request their original data and that they must supply this for peer-review to continue. Furthermore, we strongly encourage authors to make publically available the full data underpinning their articles, by depositing these in an appropriate public data repository. In conjunction with this, *Endocrine Connections* will soon require authors to include a Data Availability Statement in their article, indicating if and where the data underpinning it is publically available.

As a journal we can only do our best to identify fraud and fake science will occasionally be published. Ultimately, these deliberately falsified products taint the areas of science, the organizations to which this work is attributed and the nations that harbour these paper mills. We believe that to avoid long-term reputational harm these organizations and their national overseers need to act, for example, by establishing readily contactable offices of scientific integrity and by prosecuting those that make financial gain by falsifying science.

## Declaration of interest

The authors declare that there is no conflict of interest that could be perceived as prejudicing the impartiality of this editorial.

## Funding

This work did not receive any specific grant from any funding agency in the public, commercial, or not-for-profit sector.
